# Transcriptome Analysis of *Litsea cubeba* Floral Buds Reveals the Role of Hormones and Transcription Factors in the Differentiation Process

**DOI:** 10.1534/g3.117.300481

**Published:** 2018-02-27

**Authors:** Wenguang He, Yicun Chen, Ming Gao, Yunxiao Zhao, Zilong Xu, Pei Cao, Qiyan Zhang, Yulian Jiao, Hongsheng Li, Liwen Wu, Yangdong Wang

**Affiliations:** * State Key Laboratory of Tree Genetics and Breeding, Chinese Academy of Forestry, Beijing, China; † Research Institute of Subtropical Forestry, Chinese Academy of Forestry, Hangzhou, China; ‡ Fujian Academy of Forestry, the Key Laboratory of Timber Forest Breeding and Cultivation for Mountainous Areas in Southern China, the Key Laboratory of Forest Culture and Forest Product Processing Utilization of Fujian Province, Fuzhou, China

**Keywords:** *Litsea cubeba*, RNA sequencing, Floral bud differentiation, DEGs, Hormone, Transcription factors, Genome Report

## Abstract

**Background:**

*Litsea cubeba* (Lour.) Pers. is an important economic plant that is rich in valuable essential oil. The essential oil is often used as a raw material for perfumes, food additives, insecticides and bacteriostats. Most of the essential oil is contained in the fruit, and the quantity and quality of fruit are dependent on the flowers. To explore the molecular mechanism of floral bud differentiation, high-throughput RNA sequencing was used to detect differences in the gene expression of *L. cubeba* female and male floral buds at three differentiation stages.

**Results:**

This study obtained 160.88 Gbp of clean data that were assembled into 100,072 unigenes, and a total of 38,658 unigenes were annotated. A total of 27,521 simple sequence repeats (SSRs) were identified after scanning the assembled transcriptome, and the mono-nucleotide repeats were predominant, followed by di-nucleotide and tri-nucleotide repeats. A total of 12,559 differentially expressed genes (DEGs) were detected from the female (F) and male (M) floral bud comparisons. The gene ontology (GO) databases revealed that these DEGs were primarily contained in “metabolic processes”, “cellular processes”, and “single-organism processes”. The Kyoto Encyclopedia of Genes and Genomes (KEGG) databases suggested that the DEGs belonged to “plant hormone signal transduction” and accounted for a relatively large portion in all of these comparisons. We analyzed the expression level of plant hormone-related genes and detected the contents of several relevant plant hormones in different stages. The results revealed that the dynamic changes in each hormone content were almost consistent with the expression levels of relevant genes. The transcription factors selected from the DEGs were analyzed. Most DEGs of MADS-box were upregulated and most DEGs of bZIP were downregulated. The expression trends of the DEGs were nearly identical in female and male floral buds, and qRT-PCR analysis revealed consistency with the transcriptome data.

**Conclusions:**

We sequenced and assembled a high-quality *L. cubeba* floral bud transcriptome, and the data appeared to be well replicated (n = 3) over three developmental time points during flower development. Our study explored the changes in the contents of several plant hormones during floral bud differentiation using biochemical and molecular biology techniques, and the changes in expression levels of several flower development related transcription factors. These results revealed the role of these factors (*i.e.*, hormones and transcription factors) and may advance our understanding of their functions in flower development in *L. cubeba*.

*Litsea cubeba* (Lour.) Pers. (*Litsea*, Lauraceae) is a diecious small shrub or tree that is widely distributed in Southeast Asia (Gao *et al.* 2016). The flower, leaf and fruit of *L. cubeba* are used to extract an essential oil that possesses aromatic, antioxidant, insecticidal and antibacterial biological activities (Hwang *et al.* 2005; Amer and Mehlhorn 2006; Wang and Liu 2010; Zhang *et al.* 2012). The essential oil is often used to make natural spices, food additives, herbal medicines and insecticides and the production and export volume of Chinese essential oil has ranked first in the world for a long time (Liu and Yang 2012; Lin *et al.* 2013a). The fruit of *L. cubeba* contains the highest content of essential oil, and the quantity of the fruit depends on the quantity and quality of flowers. Therefore, flower bud differentiation is directly related to the final essential oil yield.

Studies of *L. cubeba* have mainly focused on physiology and biochemical aspects, and the molecular mechanisms associated with floral bud differentiation are rarely reported. Sequencing technology is often used to analyze the transcriptome of organisms whose genomic information is incomplete, and this technique has been improved in recent years (Yang *et al.* 2015). RNA sequencing (RNA-Seq) in plants has contributed to advances in gene expression patterns, gene functional analysis, and gene interactions (Wang *et al.* 2010). The genomic data of *L. cubeba* is not complete, and it is necessary to analyze the transcriptome for gene discovery and further functional studies. The present study, used RNA-Seq technology to analyze the gene expression information of *L. cubeba* female and male floral buds at different stages of differentiation. RNA samples from three different differentiation periods were analyzed using a high-throughput sequencing technique. Analysis of this set of transcriptome data related to bioinformatics was used to characterize floral bud transcriptional pathways during different differentiation phases of *L. cubeba*. We identified the differentially expressed genes (DEGs) that were subject to regulation during floral bud differentiation. The transcriptome sequencing of *L. cubeba* floral bud may help determine the role of various hormones and transcription factors and investigate new genes and regulatory pathways in the process of floral bud differentiation in *L. cubeba*.

## MATERIALS AND METHODS

### Plant Materials and Treatment

All female (F) and male (M) floral buds of *L. cubeba* were collected from three different stages of differentiation: (1) D1, the initial stage (5/10/2016); (2) D2, the middle stage (6/9/2016); and (3) D3, the later stage (7/29/2016) from Fuyang’s Urban Forest Park, Hangzhou, Zhejiang Province, P.R. China. In D1, the size of female floral bud was 3 ± 0.5 (mean± SD) mm long and 1 ± 0.25 mm wide, and male floral bud was similar in size. Female floral bud could be distinguished from male easily in D2, because female floral bud was smaller than male. In D2, the size of female floral bud was about 5 ± 0.5 mm long and 2 ± 0.3 mm wide, and male floral bud was about 6 ± 1 mm long and 3 ± 0.8 mm wide. In D3, this difference of size was even more apparent. The female floral bud was about 6 ± 0.45 mm long and 4.5 ± 0.5 mm wide, and male floral bud was about 8 ± 0.6 mm long and 6 ± 0.3 mm wide. Samples of each phase and gender were harvested from three trees as three independent biological replicates. All samples were immediately frozen in liquid nitrogen and stored at -80° for later use.

### Histological Observation

Verification of the differentiation period of female and male floral buds was performed using paraffin sections based on the methods of Miao (Miao *et al.* 2016). Photographs of fresh samples were taken using a stereomicroscope. Samples were placed into FAA fixative for 24 h and placed in a vacuum environment to promote fixative penetration. Samples were dehydrated in a continuous gradient of alcohol and embedded into paraffin blocks. Samples were cut into 6-10 μm using a rotary microtome. Samples were deparaffinized, stained with hematine, and mounted with neutral resins. We observed the slices and obtained photographs using an Olympus BX53 microscope (Olympus, Tokyo, Japan).

### RNA Preparation and Library Preparation for Transcriptome Sequencing

Total RNA from each sample was extracted using the RN38 EASYspin Plus Plant RNA Kit (Aidlab Biotechnologies Co., Ltd., Beijing, China). Extracted RNA was treated with RNase-free DNase I (Takara Inc., Kyoto, Japan) for 45 min at 37° to remove residual DNA. The quantity and quality of RNA were determined using gel electrophoresis and spectrophotometric analysis (Quawell Q5000, San Jose, CA, USA). The RNA integrity of these samples was assessed using the RNA Nano 6000 Assay Kit of the Agilent Bioanalyzer 2100 system (Agilent Technologies, CA, USA).

Staff at Beijing Biomarker Technologies (Beijing, China) performed isolation of mRNA, fragment interruption, cDNA synthesis, addition of adapters, PCR amplification and RNA-Seq. A total of 3 μg RNA per sample was used as input material for RNA sample preparations. mRNA-Seq libraries were constructed using the NEBNextUltra RNA Library Prep Kit for Illumina(NEB, USA). Oligo (dT) magnetic beads were used to purify mRNA from total RNA, and the mRNA was digested into fragments using NEBNext First Strand Synthesis Reaction Buffer (5X). The reverse transcriptase RNase H- and random hexamer primers were used to synthesize first-strand cDNA from the fragmented mRNA. Second strand cDNA was synthesized using DNA Polymerase I, RNase H, and dNTPs. The library of fragmented cDNA was purified using the AMPure XP system (Beckman Coulter, Beverly, USA) to select the suitable length of cDNA that would be separated using agarose gel electrophoresis and amplified using PCR. PCR products were purified (AMPure XP system) to construct the final cDNA libraries that were assessed on the Agilent Bioanalyzer 2100 system and sequenced on an Illumina HiSequation 4000 platform. Finally, the paired-end reads were generated.

### *De novo* Transcriptome Assembly and Annotation

The low quality reads, such as adaptor-only reads or reads with >10% unknown nucleotides were filtered from subsequent analyses, and the reads in which the bases of Q-score ≤10% were more than 50% were removed. After that, the high-quality clean data were used to perform *de novo* assembly. Q20, Q30, GC-content and sequence duplication level of the clean data were also calculated at this stage. Transcriptome assembly was accomplished based on the remaining high-quality clean data using Trinity software with min_kmer_cov set to 2 by default and all other parameters set to default values (Grabherr *et al.* 2011). For each library, short reads were first assembled into longer contigs by identifying their overlapping sequences. After that, different contigs from another transcript and their distance were further recognized by mapping clean reads back to the corresponding contigs based on their paired-end information, and thus the sequence of the transcripts was produced. Then, the potential transcript sequences were clustered into unigenes based on nucleotide sequence identity using the TGI Clustering tool (Pertea *et al.* 2003). The longest transcripts were eliminated based on unigenes redundancies, and the remaining unigenes were combined to produce the final assembly used for annotation.

The assembled sequences were compared against the NCBI NR, Swiss-Prot, GO, COG, KOG, eggNOG, KEGG and Pfam databases with an *E*-value ≤ 10^−5^ for the functional annotation. The Blast2GO program was used to obtain GO annotation of unigenes including molecular function, biological process, and cellular component categories.

### Simple Sequence Repeats (SSR) Screening

We selected the assembled sequences that were longer than 1kb to detect the potential SSR markers using the MIcroSAtellite (MISA) tool (http://pgrc.ipk-gatersleben.de/misa/). SSRs with motifs ranging from one to six nucleotides were analyzed. The parameters of repeat units were set for mono-, di-, tri-, tetra-, penta-, and hexa-nucleotide motifs with a minimum of 10, 6, 5, 5, 5, and 5 repeats, respectively.

### Differentially Expressed Gene (DEG) Selection

We calculated the expression levels as fragments per kilobase of transcript per million mapped reads (FPKM) for each sample. The DEGs were identified in female or male floral buds at three different differentiation stages using DESeq to detect the differentially expressed genes. Differential expression analysis of two conditions/groups was performed using the DESeq R package (1.10.1). DESeq provide statistical routines for determining differential expression in digital gene expression data using a model based on the negative binomial distribution. The resulting P values were adjusted using the Benjamini and Hochberg’s approach for controlling the false discovery rate. Genes with an adjusted P-value <0.01 & FC (Fold Change) ≥3 found by DESeq were assigned as differentially expressed. All of the DEGs were used for the COG, GO, KEGG, KOG, Pfam, Swiss-Prot, eggNOG and Nr functional annotation analyses.

### Determination of Plant Hormone Contents

Approximately 500 mg of frozen plant samples were homogenized in 4 mL of 80% methanol, extracted at 4° for 4 h, and centrifuged at 3,500 rpm for 8 min. Supernatants were collected, passed through a C-18 solid phase extraction column, and concentrated *in vacuo* to remove methanol. Na_2_HPO_4_ (200 µL, pH 9.2) was added to dissolve the sample and extracted with an equal volume of ethyl acetate three times. The ethyl acetate was discarded, and the pH was adjusted to 2.5 using 1 M HCL. Samples were extracted using 200 µL of ethyl acetate three times and collected together. The ethyl acetate was removed under vacuum conditions. IAA and ABA were dissolved in 200 µL of methanol and analyzed using ELISA after methyl esterification. ZR was measured using ELISA after dissolving in 300 µL of DBI. The detailed experimental procedure was described previously by Na *et al.* (Na *et al.* 2012).

### Analysis of Transcription Factors

PlanTFDB (http://plntfdb.bio.uni-potsdam.de/v3.0/downloads.php) was used to download the transcription factor database, and all unigenes were searched against the transcription factor database using BLASTx (E <10E^-5^). The expression levels of these transcription factors were affirmed according to the results of DEGs.

### Quantitative Real-time PCR (qRT-PCR) Verification

We subjected ten MADS-box flower development related unigenes to qRT-PCR analysis. Redundant RNA from the cDNA library preparation was used to perform reverse transcription according to the Invitrogen protocol. Suitable primers were designed using the online tool (http://frodo.wi.mit.edu/primer3/), and the amplification products were approximately 120-200bp long. The ubiquitin-conjugating enzyme E2 (*UBC*) gene was used as an internal control gene (Lin *et al.* 2013b). qRT-PCR was performed using a Power SYBR Green PCR Master Mix Kit (Applied Biosystems) to detect transcript abundance. The following experimental reaction conditions were used: denaturation at 95° for 30s, followed by 40 cycles of denaturation at 95° for 5s, annealing at 60° for 15s, and extension at 72° for 10s. All the experiments were performed with three independent replicates. The relative expression levels of the selected unigenes were calculated using the 2^-△△Ct^ method. Student’s *t*-test was used for statistical analysis. The primer sequences used in this study are listed in Table S3 in File S1.

### Data Availability

The datasets supporting the conclusions of this article are available in the NCBI Sequence Read Archive (SRA) database under accession number SRP109316. The fasta-files of the assembled transcripts are available in File S2, File S3, File S4, File S5, File S6, File S7, File S8, and File S9 and the file names are set from “*L.cubeba*. Unigene 1” to “*L.cubeba*. Unigene 8”. The fasta-files of the SSRs are available in File S10, File S11, File S12, File S13, and File S14 and the file names are set from “SSR of *L.cubeba* 1” to “SSR of *L.cubeba* 5”, and all the SSRs are described in detail in the file File S15.

## RESULTS AND DISCUSSION

### Characteristics of Different Differentiation Stages in *L.cubeba* Floral Bud

**Figure 1:**
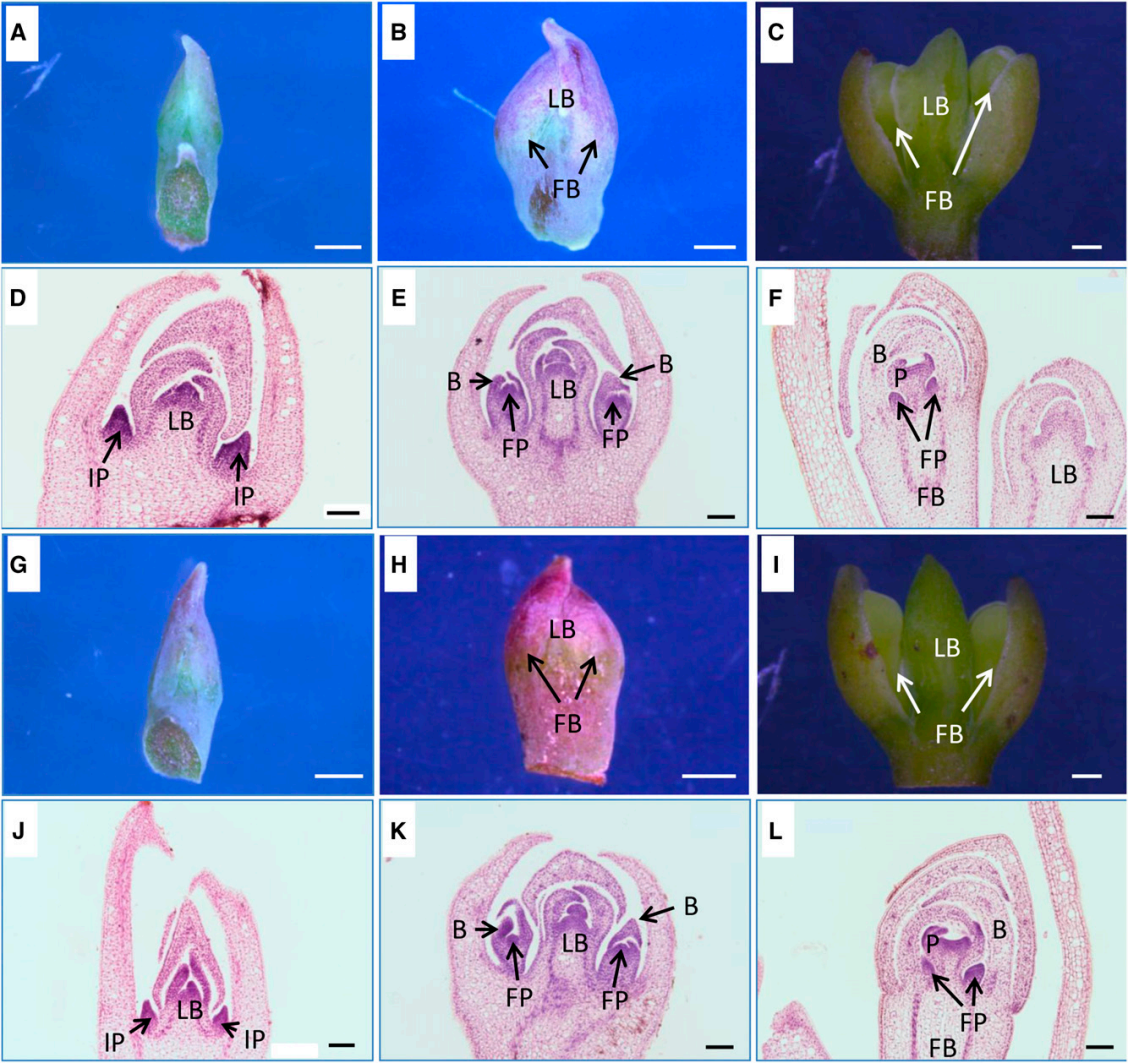
Anatomical characteristics in female and male floral bud differentiation of *L. cubeba*. Abbreviations: LB, leaf bud; FB, floral bud; IP, inflorescence primordium; FP, flower primordium; B, bract; P, perianth. (A-F) female floral bud; (G-L) male floral bud; (A, D, G, J) the inflorescence primordium begins to differentiate in the initial stage of floral bud differentiation; (B, E, H, K) the flower primordium begins to differentiate in the middle stage of floral bud differentiation; (C, F, I, L) the floral organs begin to differentiate in the later stage of floral bud differentiation. Bars = 1mm in A, B, C, G, H, and I; Bars = 100μm in D, E, F, J, K, and L.

The leaf and flower buds generally occur in the form of mixed buds and the process of floral bud differentiation is divided into three stages: D1 (the initial stage), D2 (the middle stage), and D3 (the later stage) in *L. cubeba*. In D1, the female floral bud looks very similar to the male, and we can judge whether the floral bud is female or male by the sex of the tree. In D2 and D3, the female and male floral buds are easier to distinguish because of the apparent size difference. With the development process, male floral bud is generally bigger than female. The floral bud in the initial stage may be distinguished from the leaf bud using a microscope, and the inflorescence primordium begins to differentiate (Figure 1A, D, G, and J). The floral bud grows to almost the same size as the leaf bud in the middle stage, and the flower primordium begins to differentiate in the floral bud (Figure 1B, E, H, and K). Each inflorescence of *L. cubeba* generally contains five flowers, and the flowers are arranged in a dome shape at the tip of the inflorescence axis. The flower in the middle of the other four flowers is the fastest growing (Figure 1E, F, K, and L). The floral bud enlarges significantly in the later stage, and the floral organs begin to differentiate, such as the perianth, stamen, and pistil (Figure 1C, F, I, and L).

### Illumina Sequencing and *De novo* Assembly of the Transcriptome

Eighteen RNA samples, including three biological replicates from female (F) and male (M) floral buds at three different differentiation stages (D1: the initial stage; D2: the middle stage; and D3: the later stage), were subjected to paired-end read sequencing using the Illumina HiSeq 4000 platform to obtain a comprehensive transcriptome at different developmental phases. Rigorous quality assessment and data screening generated a total of 160.88 Gbp of clean data (high-quality reads). The clean data of each sample was greater than 5.82 Gbp, and greater than 90.42% had Phred-like quality scores at the Q30 level (error <0.1%) (Table 1).

**Table 1:** Summary of Illumina transcriptome sequencing for *Litsea cubeba*

**Sample ID**	**Clean reads**	**Raw reads**	**GC(%)**	**N(%)**	**Q20(%)**	**CycleQ20(%)**	**Q30(%)**
FD1-1	38,598,058	11,378,401,194	46.18	0	95.99	100	90.86
FD1-2	40,127,970	11,847,536,088	45.91	0	96.4	100	91.62
FD1-3	35,248,975	10,401,329,460	46.34	0	96.06	100	90.98
MD1-1	46,266,563	13,700,216,204	45.95	0	95.94	100	90.54
MD1-2	39,634,935	11,720,172,148	45.7	0	96.03	100	90.75
MD1-3	28,953,078	8,551,549,176	46.34	0	95.85	100	90.42
FD2-1	30,370,299	8,973,591,738	46.54	0	96.46	100	91.75
FD2-2	28,322,757	8,361,260,110	47.05	0	95.92	100	90.69
FD2-3	22,414,438	6,618,751,272	47.18	0	96.33	100	91.52
MD2-1	28,576,174	8,445,697,070	46.5	0	96.3	100	91.43
MD2-2	27,277,997	8,055,055,324	46.85	0	96.37	100	91.61
MD2-3	21,155,473	6,251,559,524	46.76	0	96.62	100	92.07
FD3-1	24,136,985	7,124,025,324	47.25	0	96.45	100	91.74
FD3-2	24,618,235	7,281,862,830	46.76	0	96.46	100	91.75
FD3-3	22,614,916	6,693,773,474	47.16	0	96.25	100	91.37
MD3-1	24,460,477	7,216,234,650	47.06	0	96.19	100	91.24
MD3-2	26,992,258	7,973,633,670	46.74	0	96.23	100	91.34
MD3-3	34,919,660	10,282,014,812	47.01	0	96.4	100	91.63

FD1, FD2, and FD3 indicate the female floral bud in the initial, middle and later stages of differentiation, respectively. MD1, MD2, and MD3 indicate the male floral bud in the initial, middle and later stages of differentiation, respectively. -1, -2, and -3 indicate the three independent biological replicates. N means the ambiguous read. Q20 means the accuracy of base recognition is more than 99% and Q30 means the accuracy of base recognition is more than 99.9%. CycleQ20 means the cycle whose average quality score is greater than or equal to 20.

**Figure 2:**
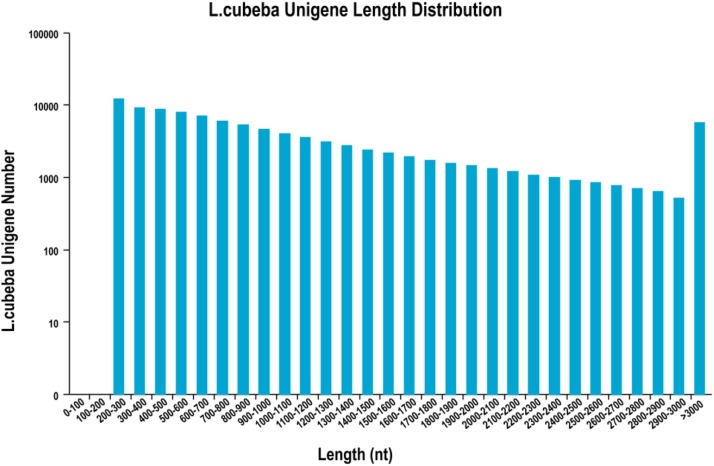
Random distribution of the assembled unigenes. The x-axis indicates the length of the unigenes. The y-axis indicates the number of unigenes.

The Trinity software *de novo* assembly program merged the clean data for floral buds at the three stages to generate the female and male *L. cubeba* transcripts data (Table 2). The two sets of transcripts were clustered into 100,072 unigenes with a mean length of 1124.17 bp, and the N50 value was 1,646 bp (Table 2). There were 69,972 unigenes of ≥500 bp and 14,667 unigenes of ≥2000 bp. Longer length unigenes enable easier functional annotation and classification. Figure 2 shows the random distribution of unigenes length, and Table 2 provides an overview of the assembled transcripts and unigenes.

**Table 2:** Length distribution of assembled transcripts and unigenes

**Length Range**	**All Unigenes**	**Transcripts of Female Floral Bud**	**Unigenes of Female Floral Bud**	**Transcripts of Male Floral Bud**	**Unigenes of Male Floral Bud**
200-300	12,262(12.25%)	30,688(11.99%)	10,649(12.89%)	36,566(15.93%)	17,272(19.08%)
300-500	17,837(17.82)	45,671(17.84%)	17,828(21.57%)	47,525(20.70%)	22,729(25.11%)
500-1000	30,783(30.76%)	70,810(27.66%)	26,645(32.24%)	60,751(26.46%)	26,740(29.54%)
1000-2000	24,522(24.50%)	64,610(25.24%)	17,998(21.78%)	50,573(22.03%)	15,553(17.18%)
2000+	14,667(14.66%)	44,187(17.26%)	9,517(11.52%)	34,142(14.87%)	8,216(9.08%)
Total Number	100,072	255,966	82,637	229,559	90,511
Total Length	112,498,390	304,528,347	82,967,468	249,275,225	79,004,082
N50 Length	1,646	1,803	1,440	1,719	1,279
Mean Length	1,124.174494	1,189.721865	1004.11	1,085.887397	872.87

### Functional Annotation and Classification of Assembled Unigenes

All unigenes were searched against eight public databases for functional annotation. Table 3 shows the integral functional annotation. Up to 38,070 and 35,559 unigenes exhibited sequence similarity to known genes when blasting to the Nr and eggNOG databases, respectively, with an *E*-value threshold of 1e^-5^. GO classification provides a strong reference for the function of unknown sequences. A total of 21,075 unigenes were annotated using GO classification and divided into three GO categories and 52 functional groups (Figure S1 in File S1). GO classification of biological processes accounted for the largest proportion, followed by cellular components and molecular function. The number of unigenes ranked in the top three categories were “metabolic process” (14,599), “cellular process” (12,207), and “catalytic activity” (11,318). A total of 11,231 unigenes were searched for COG classification (Figure S2 in File S1). There were 24 COG classes, and the largest group was “general function prediction only” (3,136), followed by “replication, recombination and repair” (1,726) and “transcription” (1,546). The smallest groups were “nuclear structures” (2) and “cell motility” (11).

**Table 3:** Functional annotation of *L. cubeba* unigenes by sequence similarity search

**Annotated_Databases**	**Annotated_Number**	**300<=length < 1000(bp)**	**length>=1000(bp)**
COG_Annotation	11,231	2,573	8,172
GO_Annotation	21,075	6,334	13,341
KEGG_Annotation	13,039	3,888	8,339
KOG_Annotation	21,970	6,171	14,420
Pfam_Annotation	26,700	6,495	19,164
Swissprot_Annotation	24,457	6,899	16,212
eggNOG_Annotation	35,559	10,457	23,112
nr_Annotation	38,070	11,591	24,187
All_Annotated	38,658	11,905	24,402

### SSR Marker Exploration

SSR is a powerful molecular marker tool for the study of biological inheritance, evolution and variety improvement. A total of 39,189 unigenes longer than 1,000 bp were selected using MISA software to identify SSR profiles in the unigenes of *Litsea cubeba*. A total of 27,521 SSRs were identified, and 1,827 unigenes had more than one SSR. Different SSR repeating units varied in length (Table 4). The mono-nucleotide repeat type made up more than half (64.43%) of the total SSRs, followed by di-nucleotide (21.86%) and tri-nucleotide (12.72%). Other types (*e.g.*, tetra-, penta-, hexa-nucleotides) had a frequency of less than 1%. The A/T type was the most frequent type in mono-nucleotide repeats and accounted for 63.68%. The AG/CT type was the most frequent type of di-nucleotide repeats and accounted for the majority proportion (15.78%), which was far greater than the other three types (AT/AT, AC/GT, and CG/GC). These results will facilitate further analyses of the genetic diversity of *L. cubeba*.

**Table 4:** Number of different SSR repeat types in the *L. cubeba* transcriptome

**Repeat type**	**Repeat number**												**Total**	**%**
	**5**	**6**	**7**	**8**	**9**	**10**	**11**	**12**	**13**	**14**	**15**	**>15**		
**Mono-nucleotide**	0	0	0	0	0	5899	3115	2225	1553	1249	1059	2633	17733	64.43
**Di-nucleotide**	0	1646	1152	1090	1218	705	189	14	0	0	0	1	6015	21.86
**Tri-nucleotide**	2124	935	408	28	3	1	0	1	0	1	0	0	3501	12.72
**Tetra-nucleotide**	188	26	0	2	0	2	0	0	0	0	0	0	218	0.79
**Penta-nucleotide**	21	1	1	0	1	0	0	0	0	0	0	0	24	0.09
**Hexa-nucleotide**	14	13	1	1	0	0	0	0	0	1	0	0	30	0.11
**Total**	2347	2621	1562	1121	1222	6607	3304	2240	1553	1251	1059	2634	27521	100
**%**	8.53	9.52	5.68	4.07	4.44	24.01	12.01	8.14	5.64	4.55	3.85	9.57	100	

### Identification of Differentially Expressed Genes (DEGs)

**Figure 3:**
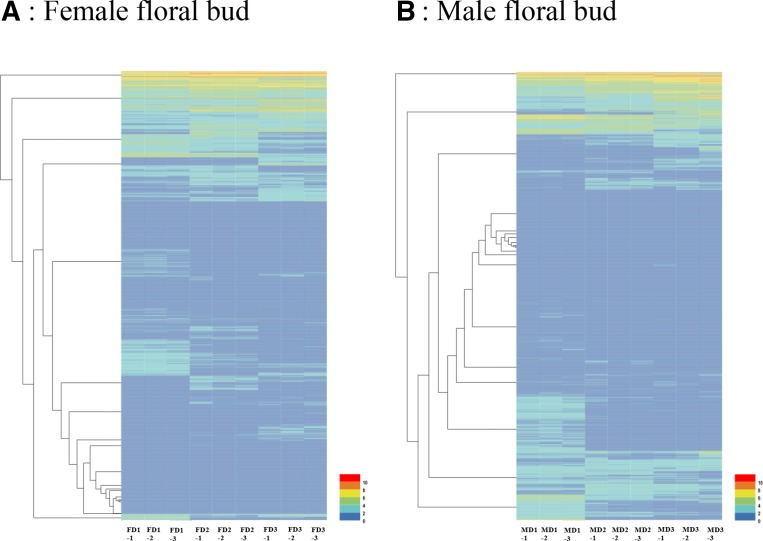
Expression profiles and cluster analysis of DEGs at different phases of *L. cubeba* female and male floral bud differentiation. FD1, FD2, and FD3 indicate the female floral bud in the initial, middle and later stages of differentiation, respectively. MD1, MD2, and MD3 indicate the male floral bud in the initial, middle and later stages of differentiation, respectively. -1, -2, and -3 indicate the three independent biological replicates.

The FPKM method was used to calculate the expression levels of unigenes in these samples. DESeq software (FDR <0.01, FC ≥3) detected a total of 12,559 DEGs from female (F) and male (M) floral bud comparisons (FD1/FD2, FD1/FD3, FD2/FD3, MD1/MD2, MD1/MD3, and MD2/MD3). FD2/FD3 or MD2/MD3 contained the smallest number of DEGs (2478 and 672) from female or male comparisons, respectively (Table 5). The results demonstrated that the differences in gene expression were greatest at the beginning of development. We analyzed the transcript abundance of genes, and the results are shown in Table 5. Compared with FD1, 1,601 and 1,848 genes were upregulated, and 1,605 and 3,136 genes were downregulated in FD2 and FD3, respectively. In FD2/FD3, 900 genes were upregulated, and 1,578 genes were downregulated. Compared with MD1, 1,296 and 1,691 genes were upregulated, and 2,524 and 4,286 genes were downregulated in MD2 and MD3, respectively. In MD2/MD3, 319 genes were upregulated, and 353 genes were downregulated. The transcript abundances of all DEGs in female or male floral buds of *L. cubeba* at different phases were clustered using hierarchical cluster analysis (Figure 3) and Principal Component Analysis (PCA) of these data obtained from all the eighteen samples was also used to visualize these differences (Figure S3 in File S1).

**Table 5:** The DEGs in different comparisons during *L. cubeba* floral bud differentiation

**DEG_Set**	**All_DEG**	**up-regulated**	**down-regulated**
**FD1/FD2**	3206	1601	1605
**FD2/FD3**	2478	900	1578
**FD1/FD3**	4984	1848	3136
**MD1/MD2**	3820	1296	2524
**MD2/MD3**	672	319	353
**MD1/MD3**	5977	1691	4286

FD1, FD2, and FD3 indicate the female floral bud in the initial, middle and later stages of differentiation, respectively. MD1, MD2, and MD3 indicate the male floral bud in the initial, middle and later stages of differentiation, respectively.

### Functional Classification of DEGs

To functionally categorize the up- and down-regulated DEGs, the GO, COG, KEGG, KOG, Pfam, Swiss-Prot, eggNOG and Nr databases were used to annotate the functions of the DEGs (Table 6). The result shows that the number of annotated DEGs in FD1/FD2 (or MD1/MD2) was greater than those in FD2/FD3 (or MD2/MD3). It suggests that the changes in the early stage of floral bud differentiation are more diverse than those in the later stage. In addition, GO enrichment of the DEGs in female and male floral bud differentiation was analyzed, and the GO annotations for these comparisons were found to be enriched for some specific GO categories (Figure S4 in File S1). Most of the DEGs in each comparison were attributed to a biological process, in which the most frequent categories of GO were “metabolic process”, “cellular process”, and “single-organism process”. It indicates that in the process of differentiation, a large number of metabolic pathways inside the floral bud tissue change, and many new substances are synthesized and the old substances are decomposed, resulting in great changes in the floral bud morphology. The GO category of cellular component revealed that the DEGs were enriched for “cell”, “cell part”, “organelle”, and “membrane” during floral bud differentiation, and the GO category of molecular function revealed that the numbers of DEGs in the two types of “catalytic activity” and “binding” were the largest. All of these results also show that during the differentiation of floral buds, many components in the cell are metabolized and a large number of enzymes play a role in the process.

**Table 6:** Functional annotations of DEGs among different comparisons during *L. cubeba* floral bud differentiation

**DEG_Set**	**Annotated**	**COG**	**GO**	**KEGG**	**KOG**	**Pfam**	**Swiss-Prot**	**eggNOG**	**Nr**
**FD1/FD2**	1825	573	981	603	959	1355	1230	1676	1795
**FD2/FD3**	1753	607	995	587	928	1369	1255	1638	1731
**FD1/FD3**	2658	741	1455	835	1306	1993	1858	2463	2615
**MD1/MD2**	2038	667	1094	716	1068	1505	1317	1881	1957
**MD2/MD3**	518	158	323	189	231	422	398	485	510
**MD1/MD3**	3002	926	1696	1049	1556	2231	2055	2801	2916

FD1, FD2, and FD3 indicate the female floral bud in the initial, middle and later stages of differentiation, respectively. MD1, MD2, and MD3 indicate the male floral bud in the initial, middle and later stages of differentiation, respectively.

According to the COG database, the DEGs were functionally clustered into 25 classifications (Figure S5 in File S1). The top three classifications in FD1/FD2 and MD1/MD2 both contained “general function prediction only” and “Replication, recombination and repair”, and FD1/FD3 and MD1/MD3 both contained “general function prediction only” and “Transcription”. These results reveals that in the whole process of floral bud differentiation, due to the dramatic increase in the number of cells, the number of genetic material such as DNA and RNA also increased significantly. The top three classifications of DEGs contained “Transcription” and “Posttranslational modification, protein turnover, chaperones” in FD2/FD3, and “Carbohydrate transport and metabolism” and “Secondary metabolites biosynthesis, transport and catabolism” in MD2/MD3, which suggests that different genes play important roles in female and male floral buds in later differentiation stages.

Different gene products coordinate with each other to perform biological functions *in vivo*, and the pathway annotation analysis of DEGs will aid further interpretations of gene function. The KEGG database is the primary public database on pathways. Table 7 shows that the top three KEGG pathways in FD1/FD2, MD1/MD2, and FD2/FD3 contained “Protein processing in endoplasmic reticulum” and “Ribosome”, which indicates that numerous proteins were synthesized at these developmental stages. However, it was strange that almost no differentially expressed genes were detected in these two pathways in MD2/MD3. Notably, “Plant hormone signal transduction” accounted for a relatively large part in all of these comparisons. These data suggest that plant hormones play a vital role in these developmental stages.

**Table 7:** KEGG pathway analysis of DEGs among different comparisons during *L. cubeba* floral bud differentiation

**KEGG Pathway**	**FD1/FD2**	**FD1/FD3**	**FD2/FD3**	**MD1/MD2**	**MD1/MD3**	**MD2/MD3**
1. Protein processing in endoplasmic reticulum	47	53	45	31	32	2
2. Ribosome	31	7	34	65	67	0
3. Carbon metabolism	30	33	25	30	32	4
4. Endocytosis	26	26	16	19	18	2
5. Spliceosome	23	21	14	23	17	0
6. Phenylpropanoid biosynthesis	23	47	31	11	42	18
7. Biosynthesis of amino acids	23	19	18	33	35	4
8. Plant hormone signal transduction	21	44	20	10	36	9
9. Plant-pathogen interaction	21	23	13	17	21	2
10. Starch and sucrose metabolism	20	35	20	23	37	10
11. Glycolysis/Gluconeogenesis	17	18	18	22	25	6
12. Amino sugar and nucleotide sugar metabolism	12	13	19	7	12	6
13. Pentose phosphate pathway	12	7	10	13	8	0
14. Phenylalanine metabolism	11	32	16	0	23	9
15. Galactose metabolism	11	16	9	6	12	0
16. Carbon fixation in photosynthetic organisms	11	15	8	14	10	1
17. Circadian rhythm-plant	11	14	0	9	9	4
18. Fatty acid metabolism	10	13	10	8	13	0
19. Cutin, suberine and wax biosynthesis	0	13	10	0	18	13
20. Glutathione metabolism	9	11	15	15	15	0

FD1, FD2, and FD3 indicate the female floral bud in the initial, middle and later stages of differentiation, respectively. MD1, MD2, and MD3 indicate the male floral bud in the initial, middle and later stages of differentiation, respectively. The top 20 enriched pathways are presented.

### Dynamic Changes in Plant Hormone Contents

Several studies demonstrated that plant flower bud differentiation was the result of the combined effects of nutrition and hormones, which are regulated by genes (Davenport 1990; Jung and Muller 2009; Ulger *et al.* 2004; van Doorn and Van Meeteren 2003). In recent years, the plant flower bud differentiation was studied from physiological and biochemical traits, molecular mechanisms, genetic control and other aspects. The results of the KEGG annotation encouraged us to measure the levels of several plant hormones in the flower buds of *L. cubeba* in different developmental stages.

### IAA (Indole-3-acetic Acid)

Plant growth hormone is primarily IAA, and endogenous IAA changes dynamically during the flower bud differentiation. Wang *et al.* .investigated the relationship between the endogenous hormones and flower bud differentiation and found that the content of IAA decreased during differentiation (Wang *et al.* 1989). Analysis of the content of endogenous hormones in litchi flower bud differentiation revealed that IAA was at a certain level in the early stage of inflorescence primordial induction and decreased after the differentiation of calyx primordium (Liang *et al.* 1987). Exogenous application of IAA promoted the formation of apple flower buds, and the use of NAA as a thinning agent promoted the flower bud differentiation of apples (Luo *et al.* 2007). Researchers generally believe that auxin may be related to the absorption of nutrients during flower bud differentiation. Ji considered that a certain level of IAA in flower buds was conducive to nutrient absorption and the promotion of flower bud differentiation (Ji 1992).

**Figure 4:**
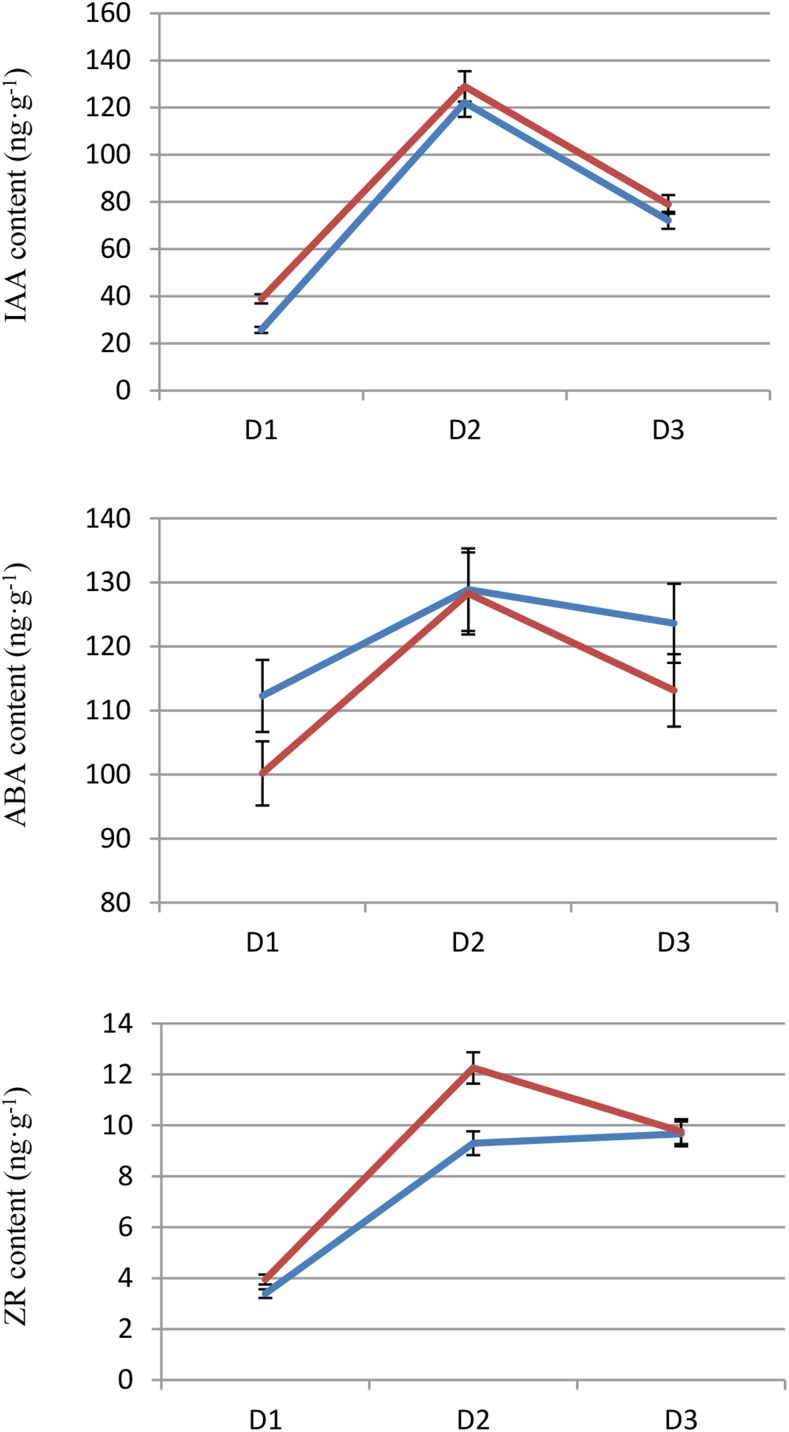
Dynamic changes of IAA, ABA, and ZR content during *L. cubeba* floral bud differentiation. X-axis represents the different differentiation stage of *L. cubeba* floral bud. D1, the initial stage; D2, the middle stage; D3, the later stage. The blue solid line represents the content of female floral bud, the red solid line represents the content of male floral bud. The data are presented as the mean ± SD of three biological replicates (each of the values is derived from the mean of three technical replicates).

Our results indicated that the content of IAA in female and male flower buds exhibited dynamic changes of an initial increase then decrease during the three differentiation periods. The IAA content increased rapidly from the D1 to D2 stage and reached its highest value in D2, followed by a reduction to approximately half in the D3 period. The IAA content in D3 remained much higher than the D1 period (Figure 4). This phenomenon suggests that a large amount of auxin was needed during the initial stage of flower bud differentiation, but this high content of auxin may exert a certain inhibitory effect in the later stage. Our transcriptome data also demonstrated that the number of upregulated IAA-related genes in the “Plant hormone signal transduction” pathway in FD1/FD2 or MD1/MD2 was significantly greater than the downregulated gene, respectively. The number of downregulated IAA-related genes was equal to or slightly greater than upregulated genes in FD2/FD3 or MD2/MD3 (Table 8).

**Table 8:** Analysis of plant hormone-related DEGs in “Plant hormone signal transduction” pathway among different comparisons during *L. cubeba* floral bud differentiation

**Plant hormone**	**FD1/FD2**		**FD1/FD3**		**FD2/FD3**		**MD1/MD2**		**MD1/MD3**		**MD2/MD3**	
	**up**	**down**	**up**	**down**	**up**	**down**	**up**	**down**	**up**	**down**	**up**	**down**
IAA-related	6	2	11	5	3	3	4	0	8	4	1	2
ABA-related	2	1	4	3	1	1	1	0	0	2	0	0
All	17	4	28	16	8	12	8	2	17	19	5	4

FD1, FD2, and FD3 indicate the female floral bud in the initial, middle and later stages of differentiation, respectively. MD1, MD2, and MD3 indicate the male floral bud in the initial, middle and later stages of differentiation, respectively. Up indicates upregulated and down indicates downregulated.

### ABA (Abscisic Acid)

The role of abscisic acid in flower bud differentiation is not conclusive. Rakngan *et al.* believed that abscisic acid promoted the flower bud differentiation of fruit trees (Rakngan *et al.* 1995). Cao *et al.* demonstrated that the ABA content increased sharply during the flower bud physiological differentiation period and was maintained at high levels after morphological differentiation, while the ABA content of leaf bud was at a low level, which indicates that the high ABA content was beneficial to flowers (Cao *et al.* 2000). Goldschmidt reported that the ABA content in the petals and styles increased from the time of flower budding to flowering and considered that this increase was related to pollination and elongation of the pollen tube. Therefore, they speculated that the increase in ABA content was conducive to the morphological differentiation of flower buds (Goldschmidt 1980).

Our analysis demonstrated that the content of ABA in female and male flower buds was at a high level in D1, and increased gradually to reach its highest value in D2, and it did not change much until D3 (Figure 4). This result suggests that ABA was favorable for flower bud differentiation, but the role of ABA may not be as significant as auxin because the content of ABA did not change much during differentiation. Our transcriptome data also revealed that the number of upregulated ABA-related genes in the “Plant hormone signal transduction” pathway in FD1/FD2 or MD1/MD2 was slightly greater than downregulated genes. The numbers of upregulated and downregulated ABA-related genes were very few and identical in FD2/FD3 or MD2/MD3 (Table 8).

### CTK (Cytokinin)

Cytokinin can promote plant flower bud differentiation. Luckwill demonstrated that cytokinin in xylem played a very important role during a critical period of apple flower bud differentiation (Luckwill 1980). Li *et al.* reported that the level of ZR (Zeatin riboside) increased during the physiological differentiation of apple flower bud, which indicated that ZR was beneficial to flower bud differentiation (Li *et al.* 2000). Li *et al.* demonstrated that treatment of flower buds with BA (benzyl adenine) after morphological differentiation significantly promoted the development of flower organs and increased the number of flowers the next year (Li and Deng 1992). Cytokinin activates the cell division of flower buds to promote flower development (Tamas 1995). Our study demonstrated that the content of ZR in female and male flower buds increased approximately 2 times from D1 to D2 then changed slightly, but this change was not significant until D3 (Figure 4). This result suggests that the process of flower bud differentiation requires a number of cytokinins, that is, cytokinin plays an important role in differentiation, perhaps activation of the division of flower bud cells.

### Ethylene

Flowers of most angiosperms are bisexual with only 10% being unisexual and sex determination is a developmental process that leads to unisexual flower. Ethylene has a significant impact on the sex determination of plants, with high concentration inducing female flower differentiation and lower concentration inducing male (Boualem *et al.* 2015). However, researchers have not yet clarified the molecular mechanism by which ethylene suppresses male flower differentiation but promotes female. In this study, we found that the expression level of most ethylene-responsive transcription factors in male floral buds was higher than that in female floral buds in D1. In D2, Only a few ethylene-responsive transcription factors expressed slightly higher in male floral buds than in female floral buds. However, in D3, the expression level of most ethylene-responsive transcription factors in female floral buds was higher than that in male floral buds, and flower organs began to differentiate at this time (Table S1). These results may indicate that, with the progress of floral bud differentiation, the content of ethylene will change to be higher in the female floral bud than in male. This may provide some evidence that ethylene can promote the differentiation of female flowers. Further research on these ethylene-responsive transcription factors to find their downstream target genes may be able to screen out the critical factors for flower sex differentiation and elucidate the relevant molecular mechanism.

### Major Transcription Factors of Floral Bud Transcriptome

Genes associated with flower development were cloned and analyzed for function over the past decade. Transcription factors are primary regulators that control gene clusters (Zhang *et al.* 2011). Transcription factors generally regulate the expression level of target genes via binding to the *cis*-acting element in the promoter region. Some data indicate that many transcription factors, such as MADS-box, Zinc_finger, MYB and bZIP gene families, regulate flower development (Abe *et al.* 2005; Chiou and Yeh 2008; Chuang *et al.* 1999; Sawa *et al.* 1999; Albert *et al.* 2011; Kranz *et al.* 1998; Payne *et al.* 2004; Theißen and Saedler 2001). These transcription factors were selected from the transcriptome data to evaluate their association with flower bud differentiation.

### The MADS-box Family

Numerous studies have demonstrated that MADS-box genes play a part in various steps of the transition from vegetative to reproductive growth. *AP1*, *AGL2*, *AGL4*, and *AGL9* from Arabidopsis are involved in the regulation of floral meristem initiation and development (Mandel *et al.* 1992; Flanagan and Ma 1994; Savidge *et al.* 1995; Mandel and Yanofsky 1998). *OsMADS1* from rice regulates differentiation of the inner floral organ, and *FBP2* from petunia is involved in floral development (Prasad *et al.* 2005; Angenent *et al.* 1994). We identified 39 MADS-box transcription factors among these unigenes, including 23 DEGs (Table S2). The expression trends of the DEGs were nearly identical in female and male floral bud transcriptomes of the three differentiation stages. Seventeen DEGs were upregulated, and 6 DEGs were downregulated. *074460*, *048758*, and *085161* all exhibited low expression levels in D1 but very high levels in D3, which were upregulated greater than one hundred times. These data indicate that these three genes play an important role in the late stage of flower bud differentiation. The expression level of *057147* was similar and quite high in D1 and D2 but downregulated to one-tenth in D3, which suggests that it was greatly inhibited in the late stage of flower bud differentiation. The proportion of DEGs in MADS-box was the highest compared with other transcription factors, and most of the DEGs were upregulated from D1 to D3. These data indicate that MADS-box transcription factors are very important in the process of flower bud differentiation.

### The Zinc_finger Family

Zinc_finger transcription factors regulate flower development. The *SUPERMAN* (*SUP*) gene in *Arabidopsis* is necessary for the proper spatial development of floral organs (Sakai *et al.* 1995). Another gene encoding a protein with a zinc_finger domain, *FILAMENTOUS FLOWER*, is responsible for the normal development of floral organs and the maintenance of meristem activity (Sawa *et al.* 1999). *RABBIT EARS* (*RBE*) is the transcription factor that decides the development of the second whorl organs independently of the organ identity (Takeda *et al.* 2004). Functional analyses of *ZINC FINGER PROTEIN2* (*AtZFP2*) revealed that it participated in processes that influence the shedding of floral organs (Cai and Lashbrook 2008). In total, 1100 zinc_finger transcription factors, including 88 DEGs, were identified in our transcriptome data. Forty-six DEGs were upregulated, and 42 DEGs were downregulated (Table S2). The percent of DEGs in zinc_finger protein was generally much lower than that in MADS-box, and the expression of DEGs differed slightly more in zinc_finger genes than in MADS-box.

### The MYB Family

MYB-related proteins were identified in nearly all eukaryotes, and plants contain numerous myb transcription factors that are involved in diverse gene regulation. Relevant studies confirmed that myb genes play an important role in flower development. For example, OsGAMYB is important for floral organ development and essential for pollen development, and other *GAMYB*-like genes mediate GA signaling in growth and flowering responses (Kaneko *et al.* 2004; Gocal *et al.* 2001). There were 434 myb genes in our transcriptome database, and 53 were DEGs. Twenty-four DEGs were upregulated, and 29 DEGs were downregulated (Table S2).

### The bZIP Family

The bZIP proteins are present in a variety of plants and participate in various biological processes, including flower development. The excessive expression of pepper *CAbZIP1* in *Arabidopsis* delayed plant growth and reduced the number of petals (Lee *et al.* 2006). The decrease in *BZI-1* gene expression level in tobacco resulted in smaller flowers and affected the development of petals and stamens (Strathmann *et al.* 2001). Zou *et al.* believed that *OsABI5* regulated the fertility of pollen in rice (Zou *et al.* 2008). Seventy-six bZIP transcription factors were found in our study, and 9 were DEGs. Most DEGs (7) were downregulated (Table S2). This result suggests that most bZIP transcription factors exhibit a negative regulatory effect in flower bud differentiation.

### Expression Analysis of Genes of Interest

**Figure 5:**
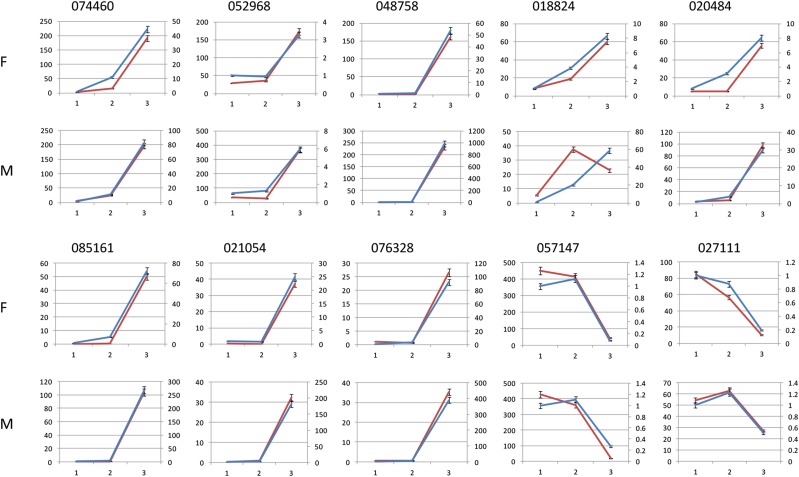
qRT-PCR analysis of 10 DEGs during *L. cubeba* floral bud differentiation. X-axis represents the different differentiation stage of *L. cubeba* floral bud. 1, the initial stage; 2, the middle stage; 3, the later stage. The left Y-axis represents the expression level of genes according to the FPKM value. The right Y-axis represents the expression level (2^-△△Ct^ value) of genes. The red solid line represents the expression level of genes according to the FPKM value, the blue solid line represents the expression level (2^-△△Ct^ value) of genes using qRT-PCR. The qRT-PCR data are presented as the mean ± SD of three biological replicates (each of the values is derived from the mean of three technical replicates). F indicates female floral bud and M indicates male floral bud.

This study selected 8 upregulated (*074460*, *052968*, *048758*, *018824*, *020484*, *085161*, *021054*, and *076328*) and 2 downregulated (*057147* and *027111*) DEGs of MADS-box for qRT-PCR validation. PCR amplification revealed that all qRT-PCR primers used produced only single fragments of the expected lengths. All fragments were sequenced, and the valid primers were used in the qRT-PCR experiment. The transcript abundances of these DEGs were calculated using log_2_ ‘relative FPKM’. The results (Figure 5) of the qRT-PCR analysis of most selected genes were consistent with the transcriptome data.

### CONCLUSIONS

This study used transcriptome data for the first time to analyze differences in gene expression in female and male floral buds of *L. cubeba* at different differentiation stages. A total of 38,658 unigenes were annotated, and 12,559 DEGs were detected in different comparisons. This large amount of transcriptome data provide a reference for future gene cloning. A variety of plant hormones and transcription factors regulate the process of flower development, and we focused on the expression trend of several plant hormone-related regulatory genes and transcription factors. These results will facilitate future analyses of the role of these genes in flower development. We identified 27,521 SSRs that may serve as an effective tool for future plant breeding.

## Supplementary Material

Supplemental Material is available online at www.g3journal.org/lookup/suppl/doi:10.1534/g3.117.300481/-/DC1.
