# Supplementary material for: Transcriptome Analysis of Litsea cubeba Floral Buds Reveals the Role of Hormones and Transcription Factors in the Differentiation Process
Source: G3 (Bethesda). 2018 Feb 27;8(4):1103–14. doi: 10.1534/g3.117.300481 (PMC5873901; doi:10.1534/g3.117.300481)
Supplement: Supplementary file 1 [file 1103FileS1.docx]

**
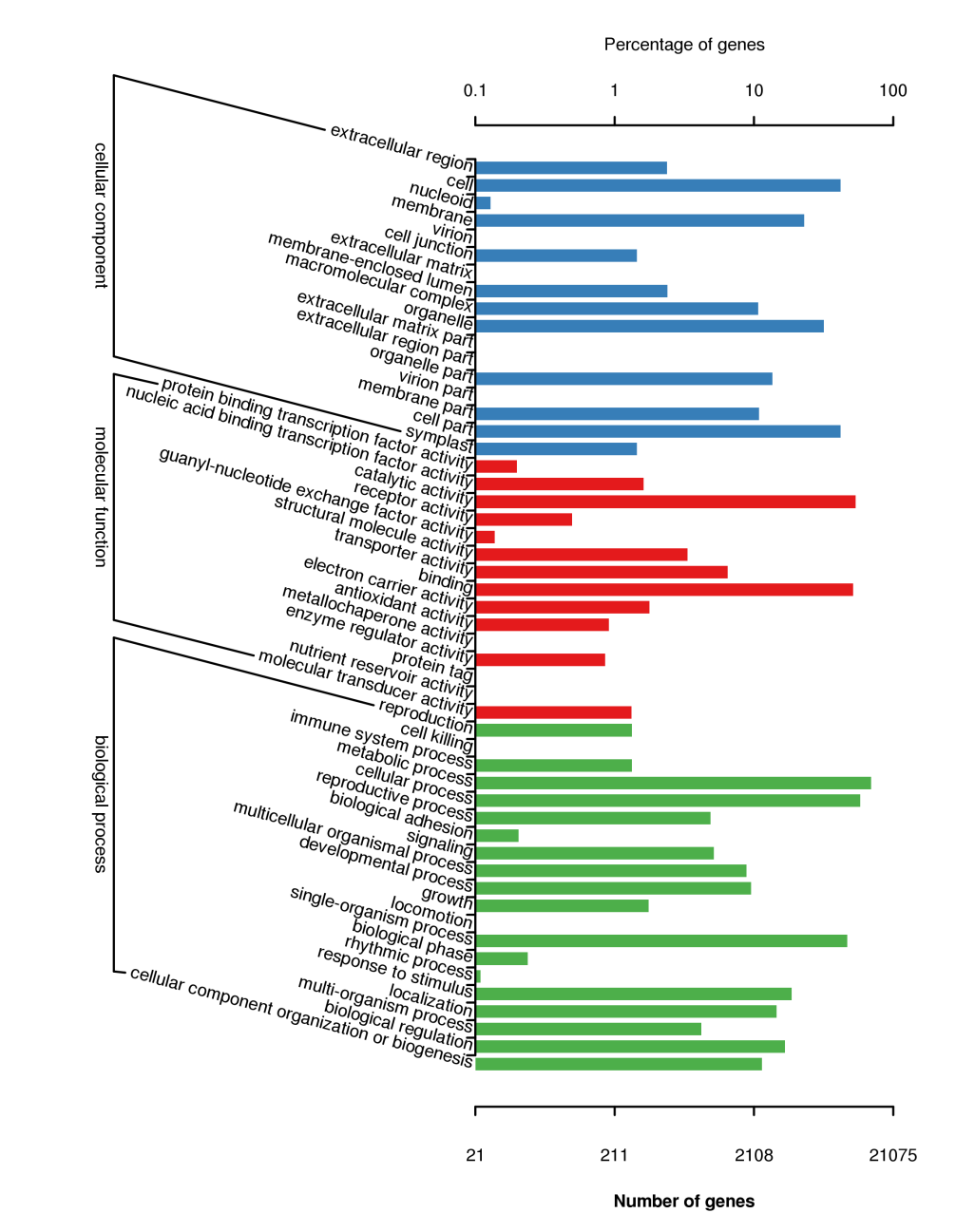
**

**Supplementary Figure 1 Functional annotation of assembled sequences based on gene ontology (GO) categorization.**

**
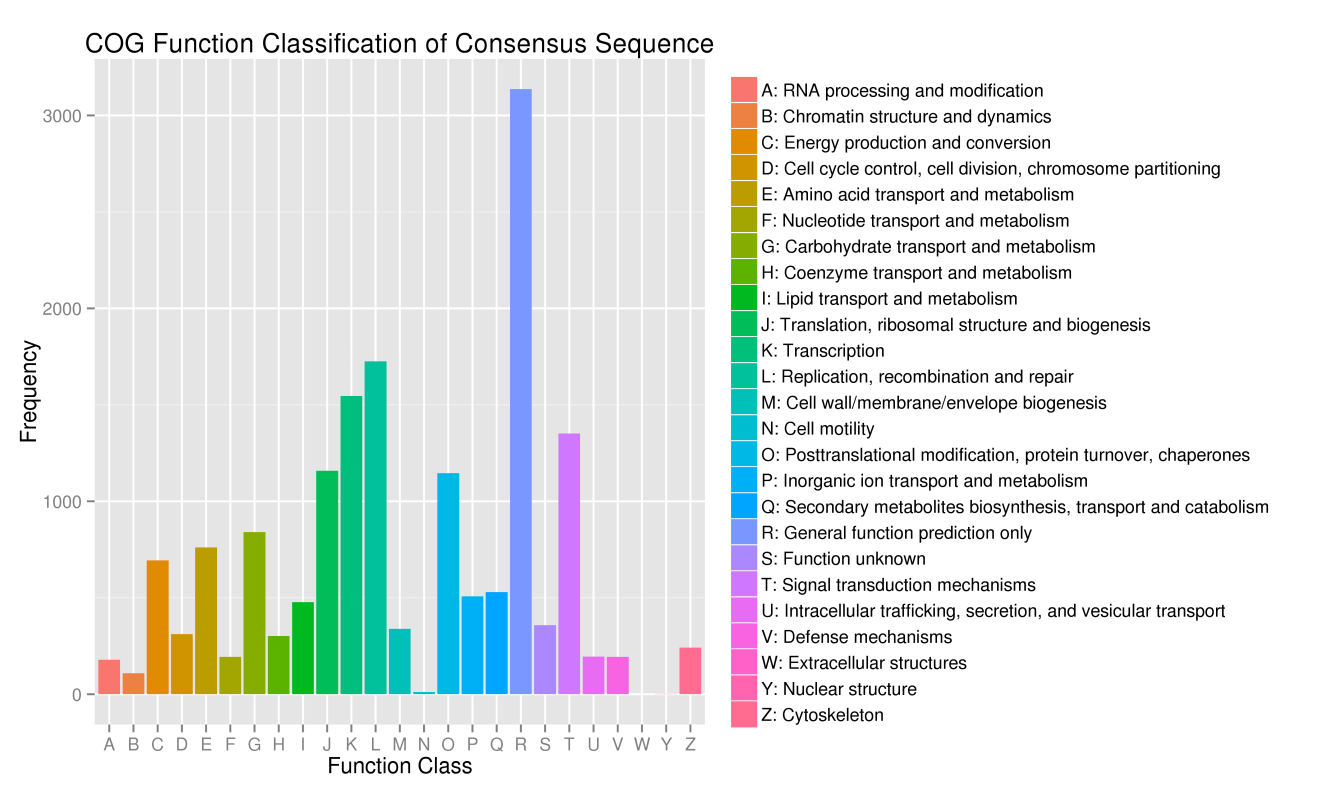
**

**Supplementary Figure 2 Clusters of orthologous group (COG) classification.**

**
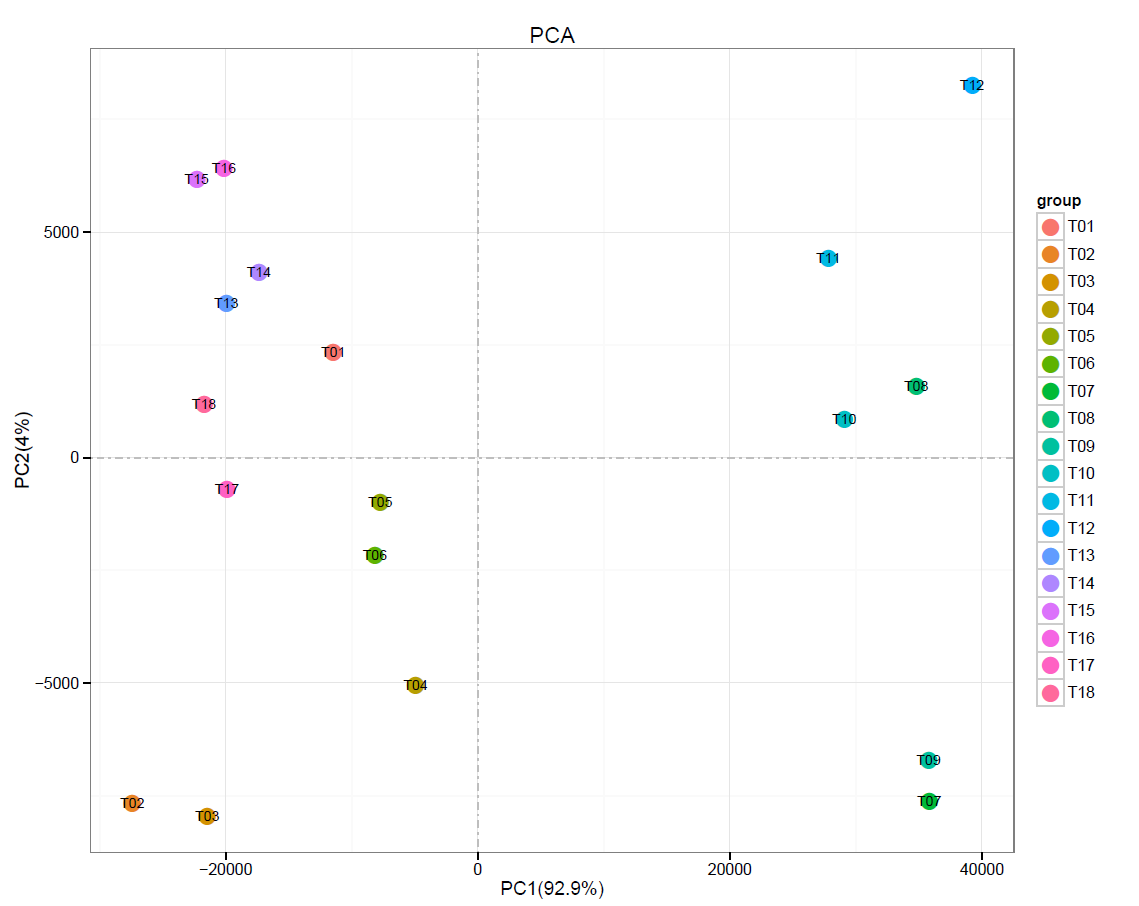
**

**Supplementary Figure 3 Principal component analysis (PCA) of transcriptome data.** T13, T15, and T17 indicate the three biological replicates of FD1 (FD1-1, FD1-2, and FD1-3). T14, T16, and T18 indicate the three biological replicates of MD1 (MD1-1, MD1-2, and MD1-3). T01, T02, and T03 indicate the three biological replicates of FD2 (FD2-1, FD2-2, and FD2-3). T04, T05, and T06 indicate the three biological replicates of MD2 (MD2-1, MD2-2, and MD2-3). T07, T08, and T09 indicate the three biological replicates of FD3 (FD3-1, FD3-2, and FD3-3). T10, T11, and T12 indicate the three biological replicates of MD3 (MD3-1, MD3-2, and MD3-3).

**Supplementary Figure 4 Gene ontology (GO) enrichment analysis of DEGs among different comparisons during *L. cubeba* floral bud differentiation.** FD1, FD2, and FD3 indicate the female floral bud in the initial, middle and later stages of differentiation, respectively. MD1, MD2, and MD3 indicate the male floral bud in the initial, middle and later stages of differentiation, respectively. The x-axis shows the Gene Ontology (GO) classification of *L. cubeba* transcriptome. The y-axis on the right show the number of genes in category, and that on the left show the percentage of a specific category of genes in that main category. The upper number of the y-axis on the right means the number of DEG unigenes in a specific category and the lower one means the number of all unigenes in a specific category.

**Supplementary Figure 5 Clusters of orthologous groups classification of DEGs among different comparisons during *L. cubeba* floral bud differentiation.** FD1, FD2, and FD3 indicate the female floral bud in the initial, middle and later stages of differentiation, respectively. MD1, MD2, and MD3 indicate the male floral bud in the initial, middle and later stages of differentiation, respectively.

**Supplementary Table 1 Major ethylene-responsive transcription factor identified in the DEG database.**

**Supplementary Table 2 Major transcription factors identified in the DEG database.**

**Supplementary Table 3 Primers used for qRT-PCR analysis.**

| **Gene ID** | **Forward (5’-3’)** | **Reverse (5’-3’)** |
| --- | --- | --- |
| ***074460*** | TAACAAAGACCCAGTTCGTGC | TTCCCACGAGAGTTGAAGGTT |
| ***052968*** | CCAAAGGAACTCATCCCAATC | TCATCCTCCAAGTTTCTTCCAT |
| ***048758*** | GACCGTTATCAACAAGTCTCCG | TGATACTTTCGTTCACGGACA |
| ***018824*** | CTGTTCTATGTGACGCTGAGGT | CTATTGTTCTTGGATGCCTGTC |
| ***020484*** | GGAATCTGGAGCAGTAGTTGG | CATCCCTGAATGAAGTTGCTCT |
| ***085161*** | GAGCCTAAGAAGAAGGTTGGAAG | CGATTGTGATTTGATTAGACTGGT |
| ***021054*** | ACCAGAAGGCATCAGGGAAT | CCCTTCAAGTGCCTTAGTTCG |
| ***076328*** | TGAAGATTGTCCGTGAACGAAAG | ATCATCTTTTCCTTCCTGTGCTCGC |
| ***057147*** | TCGCACTGAGCTGAGCAAAC | GCAGGGAGGAAATGTAAACT |
| ***027111*** | AAAAGGCACTACAGAAGTCCAAG | GTGTCGCTCTTGTTGTCCCT |
| ***UBC*** | TGTGTGTGTGTGTGTGTGTCC | CCTTTCTCCACGGTCTTCAA |
